# Diffusion in Sephadex Gel Structures: Time Dependency Revealed by Multi-Sequence Acquisition over a Broad Diffusion Time Range

**DOI:** 10.3390/math9141688

**Published:** 2021-07-19

**Authors:** Guangyu Dan, Weiguo Li, Zheng Zhong, Kaibao Sun, Qingfei Luo, Richard L. Magin, Xiaohong Joe Zhou, M. Muge Karaman

**Affiliations:** 1 Center for Magnetic Resonance Research, University of Illinois at Chicago, IL 60612, USA; 2 Department of Bioengineering, University of Illinois at Chicago, IL 60607, USA; 3 Research Resource Center, University of Illinois at Chicago, IL 60612, USA; 4 Department of Radiology, Northwestern University, IL, 60611, USA; 5 Department of Radiology, University of Illinois College of Medicine, IL 60612, USA; 6 Department of Neurosurgery, University of Illinois College of Medicine, IL 60612, USA

**Keywords:** continuous-time random-walk, diffusion MRI, diffusion time, Sephadex gel phantom

## Abstract

It has been increasingly reported that in biological tissues diffusion-weighted MRI signal attenuation deviates from mono-exponential decay, especially at high *b*-values. A number of diffusion models have been proposed to characterize this non-Gaussian diffusion behavior. One of these models is the continuous-time random-walk (CTRW) model, which introduces two new parameters: a fractional order time derivative *α* and a fractional order spatial derivative *β*. These new parameters have been linked to intravoxel diffusion heterogeneities in time and space, respectively, and are believed to depend on diffusion times. Studies on this time dependency are limited, largely because the diffusion time cannot vary over a board range in a conventional spin-echo echo-planar imaging sequence due to the accompanying T2 decays. In this study, we investigated the time-dependency of the CTRW model in Sephadex gel phantoms across a broad diffusion time range by employing oscillating-gradient spin-echo, pulsed-gradient spin-echo, and pulsed-gradient stimulated echo sequences. We also performed Monte Carlo simulations to help understand our experimental results. It was observed that the diffusion process fell into the Gaussian regime at extremely short diffusion times whereas it exhibited a strong time dependency in the CTRW parameters at longer diffusion times.

## 1. Introduction

Using water diffusion as a probe, diffusion-weighted MRI (DW-MRI) has become a promising technique to reveal the underlying micrometer-scale structural properties in millimeter-resolution MR images [1,2]. In DW-MRI, two diffusion gradient lobes are employed to dephase and rephase spins, respectively. The displacement of water molecules is quantified over a given time period, known as effective diffusion time (Δ_eff_), which is constrained by the separation and duration of diffusion gradient lobes. For water molecules that diffuse during Δ_eff_, the varying degree of spatial dislocation results in a phase dispersion (Φ) of the magnetization. The probabilistic distribution function (PDF) of the net displacement of diffusing water molecules is related to a probability distribution of Φ, which leads to signal attenuation in an MRI measurement [3,4].

It is widely accepted that the probability distribution of molecular displacement is Gaussian in an isotropic, homogeneous, and unrestricted medium. In that case, the second moment of the distribution, or mean squared displacement (MSD), scales linearly with diffusion time: <x^2^> ~ t [5]. In the presence of restricting barriers in complex materials, however, the probability distribution of molecular displacement no longer follows Gaussian distribution. The MSD in the non-Gaussian diffusion case can be characterized as a function of intrinsic diffusion coefficient, restrictive geometry, and diffusion time [6]. One way of characterizing the non-Gaussian diffusion behavior is to employ the continuous time random walk (CTRW) theory, in which the MSD can be expressed by a composite power law as: <x^2^> ~ t^2*α*/*β*^, where *α* and *β* are the fractional order time and space derivatives, respectively, in fractionalized Fick’s second law [7,8]. This generalized description enables the CTRW model to provide a more realistic description of the complex diffusion pattern in biological tissues [8].

In parallel to the development of non-Gaussian diffusion models, it has been increasingly recognized that diffusion parameters derived from various diffusion models exhibit dependence on diffusion time. Pyatigorskaya el al. [9] and Aggarwal et al. [10] observed substantial time dependency in diffusion kurtosis imaging (DKI) in the mouse brain while noticeable time dependency of intravoxel incoherent motion (IVIM) model was also reported by Wu et al. [11] in a flow phantom and mouse brain. Varying diffusion time enables exploration of the interaction between diffusing water molecules and the surrounding environment at different spatial scales [12], providing a new degree of freedom to estimate parameters that are related to the underlying tissue microstructures [12,13]. Conventional spin-echo-based DWI sequences have limited ability to vary diffusion time. On one hand, a longer diffusion time results in substantial increase in echo time, leading to signal loss due to T2 decay. On the other hand, a shorter diffusion time reduces the *b*-value, leading to inadequate diffusion-weighting. Therefore, investigation of diffusion time dependency in DWI-MRI over a broad range requires alternative pulse sequences to lengthen or shorten the effective diffusion time. Although previous studies investigated the time dependency of the CTRW parameters in the intermediate to long diffusion time range [8,14,15], the time dependency of the CTRW model at short diffusion time remains unexplored. Furthermore, the consistency of the CTRW parameters under different pulse sequences has not been well studied.

Sephadex gels are structurally heterogeneous swollen polymers with relatively uniform pore size [16], forming an ideal test bed to mimic the diffusion environment in complex biological tissues. The multi-compartment Sephadex beads provide different physical environments, such as restricted diffusion environment within the bead, free liquid space outside the bead, and open pores for water molecules to diffuse through. In this complex multi-compartment environment, water molecules move freely at short diffusion times while they experience hinderance or restriction when interacting with polymer structures at longer diffusion times. Sephadex gel phantoms have been widely used for validating advanced diffusion models, such as the CTRW model’s predecessor–fractional order calculus model [7,14,17].

In this study, we investigated the diffusion time dependency of the CTRW model by employing oscillating-gradient spin-echo (OGSE), pulsed-gradient spin-echo (PGSE), and pulsed-gradient stimulated echo (PGSTE) pulse sequences. Collectively, these sequences enabled investigation of a broad range of diffusion times, spanning from short-, intermediate-, to long-time regime. Two series of Sephadex gels, each with different pore size or bead diameters, were selected as the experimental material to mimic the tissue environment. In addition, Monte Carlo simulations were performed to help understand the experimental results.

## 2. Theory

According to the simple random walk (RW) theory, the one-dimensional Brownian motion of a diffusing particle in a homogeneous and isotropic environment can be described by a second-order partial differential equation,

(1) $$\frac{\partial P(x,t)}{\partial t}=D\frac{\partial^{2}P(x,t)}{\partial {\left|x\right|}^{2}},$$

where *P*(*x*,*t*) is one-dimensional Brownian motion of a diffusing particle and *D* is the diffusion coefficient. The solution to Equation (1) yields Gaussian distribution of the displacement where the MSD is proportional to diffusion time *t*, 〈x^2^(*t*)〉 ~ *t* [8].

In the context of the CTRW theory, where jump length and jump waiting time follow asymptotic power law distributions, the one-dimensional anomalous motion of a diffusing particle in a heterogenous environment can be described with a dual space-time fractional order diffusion equation of the form [7,18–20],

(2) D0CtαPx,t=Dα,β∂βP(x,t)∂xβ,

where *D*_*α,β*_ is the anomalous diffusion coefficient (in mm^β^/s^α^), D0Ctα is the αth (0 < α ≤ 1) fractional order time derivative in the Caputo form, given as [21]:

(3) D0Ctαft=1Γ(n−α)∫0tt−τn−α−1ddτnf(τ)dτ.

$\frac{\partial^{\beta }P(x,t)}{\partial {\left|x\right|}^{\beta }}$ in Equation (2) is the *β*th (0 < *β* ≤ 2) fractional order space derivative in the Riesz form. For *n*−1 < *β* < *n* and the finite interval 0 ≤ *x* ≤ *L*, the Riesz fractional operator is defined as [22]:

(4) ∂βu(x,t)∂xβ=−12cos⁡πβ2Dxβ+DLβx0ux,t,

where

(5) Dxβ0ux,t=1Γ(n−β)∂n∂xn∫0xuξ,tdξ(x−ξ)β+1−n

(6) DLβxux,t=1Γ(n−β)∂n∂xn∫xLuξ,tdξ(x−ξ)β+1−n.

With the representation in Equation (2), the MSD can be represented by 〈x^2^(*t*)〉 ~ *t*^(2*α*/*β*)^. When *α* = 1 and *β* = 2, this formalism is reduced to the classical Gaussian expression. In comparison, when 2*α* > *β* or 2*α* < *β*, the anomalous diffusion process is referred to as super-diffusive or sub-diffusive [7,17] respectively, and when 2*α* = *β*, the non-Gaussian dynamics is described as quasi-diffusion [23].

For a Stejskal–Tanner diffusion gradient pulse, the solution to Equation (2) can be described as:

(7) $$\frac{S}{S_{0}}=P\left(q,\Delta_{eff}\right)=E_{\alpha }\left(-D_{\alpha ,\beta }q^{\beta }{\Delta_{eff}}^{\alpha }\right).$$

In Equation (7), *S*_*0*_ is the signal intensity without diffusion weighting and *S* is the signal intensity at *q* and Δ_eff,_ where *q* = *γG*_*diff*_*δ* and Δ_eff_ = Δ – *δ*/3 in which *γ* is the gyrometric ratio, *G*_*diff*_ is the diffusion gradient amplitude, *δ* is the diffusion gradient pulse width, and Δ is the diffusion gradient separation. *E*_*α*_ is a single-parameter Mittag–Leffler function [8]. For other diffusion gradient waveforms, expressions analogous to Equation (7) can be derived in reference of the methods described in [24,25].

## 3. Methods

### 3.1. Sephadex Gel Phantom Preparation:

In this study, we used two series of Sephadex gels (GE Healthcare) which were characterized by two numbers (e.g., G25–50), where the first number indicates the macromolecular exclusion limit (in kDaltons, positively correlated with internal pore size) and the second denotes the maximum dry bead diameter (in microns). The first series of gels (G25–50, G50–50, G75–50) had the same dry bead diameter of 50 microns, but with increased macromolecular exclusion limit. The second series of gels (G50–50, G50–80, G50–150) had the same internal pore size of 50 kDaltons, but increased bead diameter. The first series of gels were designed to mimic varying microstructure permeability while the second series to simulate varying microstructural scale.

Sephadex gel phantoms were prepared by gently pouring excess distilled water into the dry power gel in a cylindrical test tube (inner diameter = 13.5 mm) at room temperature; and mixed evenly by using a vortex shaker. The slurry was allowed to settle under the influence of gravity; and the residual water was removed by pipette before sealing.

### 3.2. Data Acquisition:

**Figure 1.**
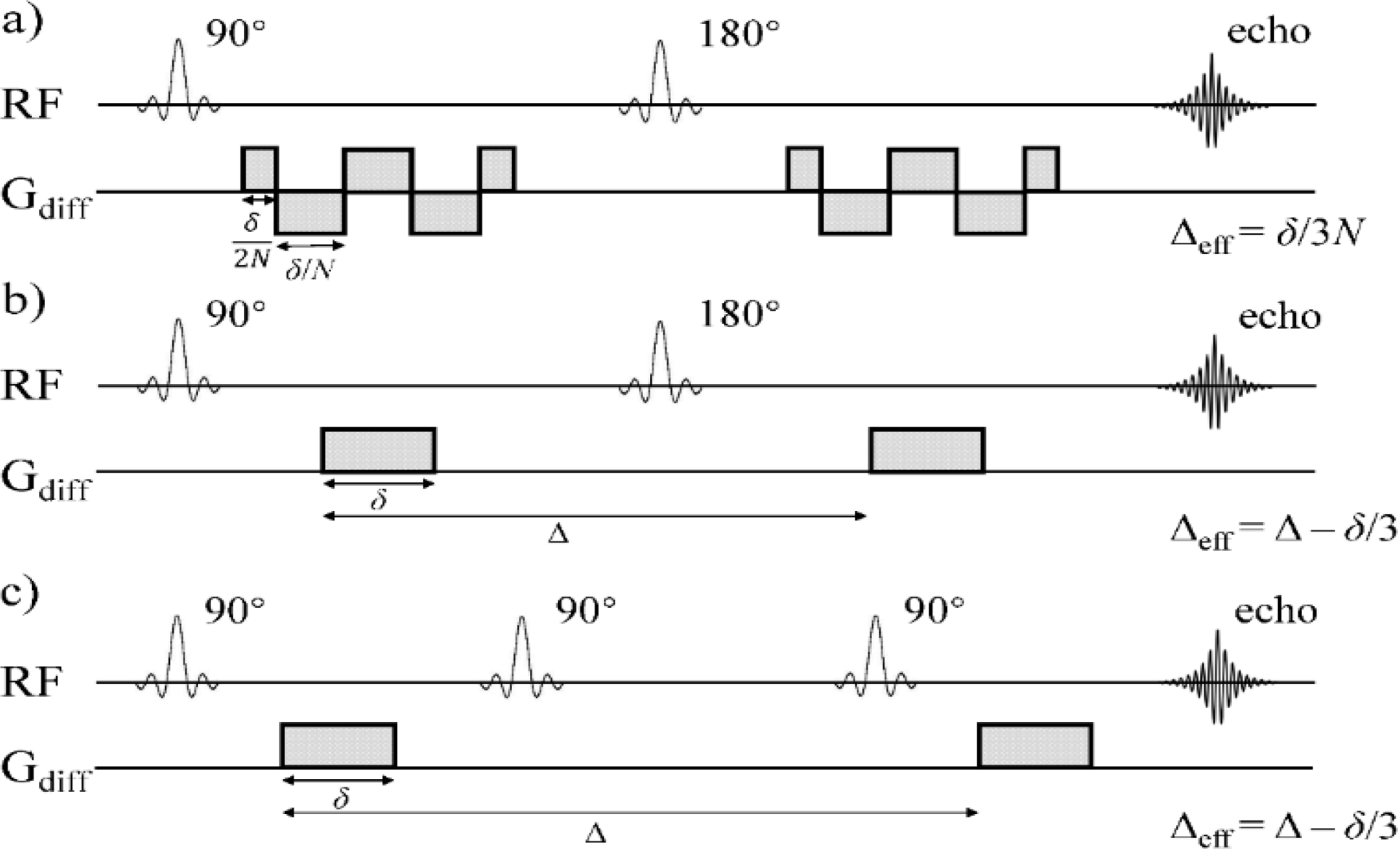
Pulse sequences employed in this study. (**a**) Cosine-trapezoid OGSE where *N* is the number of half oscillation period and *δ* is the total waveform duration (*N* = 4 in the sequence diagram). (**b**) PGSE where *δ* is the diffusion lobe duration and Δ is the diffusion lobe separation. (**c**) PGSTE where *δ* and Δ are defined similarly as in (**b**).

The experiments were performed on an Agilent 9.4 T small animal MRI scanner with a maximum gradient of 1000 mT/m. Prepared Sephadex gels were scanned on the scanner at the room temperature of 22 °C. As illustrated in Figure 1, three different DW pulse sequences were employed to investigate the diffusion time dependency of the CTRW model parameters across a broad range of diffusion times.

#### (I) Customized cosine-trapezoid OGSE sequence:

OGSE sequence enables a short effective diffusion time by periodically varying the polarity of diffusion encoding gradients. Cosine-trapezoid OGSE waveforms start and end with a quarter-period lobe. Δ_eff_ and *b*-value of the cosine-trapezoid OGSE sequence are given by [26]:

(8) $$\Delta_{\text{eff}}=\delta /\left(3N\right)$$

(9) $$b=\frac{\gamma^{2}{G_{diff}}^{2}\delta^{3}}{6N^{2}}$$

where *δ* is the total waveform duration and *N* is the number of half oscillation periods (Figure 1a). Three OGSE acquisitions were performed with a constant *δ* of 30 ms while *N* was set to 6, 4, and 2, resulting in Δ_eff_ values of 1.67, 2.5, and 5 ms, respectively.

#### (II) PGSE sequence:

The effective diffusion time, Δ_eff_, under a Stejskal–Tanner diffusion sensitizing gradient pair in a PGSE sequence is given by [3]:

(10) $$\Delta_{\text{eff}}=\Delta -\delta /3$$

(11) $$b=\gamma^{2}{G_{diff}}^{2}\delta^{2}\Delta_{eff}$$

The PGSE experiments were performed with Δ = 11 ms and 35 ms, and *δ* = 2.5 ms, resulting in Δ_eff_ values of 10.17 ms and 34.17 ms, respectively (Figure 1b).

#### (III) *PGSTE sequence*:

With the same Stejskal–Tanner diffusion sensitizing gradient pair as in PGSE, a PGSTE sequence achieves long diffusion time by taking advantage of the slower T1 relaxation rate during mixing time (TM). The DW-MRI data were acquired with an identical Δ as in the PGSE sequence (35 ms), and three longer Δ values (80 ms, 100 ms, and 150 ms). The corresponding Δ_eff_ values were 34.17, 59.17, 99.17, and 149.17 ms, respectively (Figure 1c).

At each diffusion time, DW images with 11 *b*-values (0, 25, 100, 225, 400, 625, 900, 1225, 1600, 2025, and 2500 s/mm^2^) were acquired from the Sephadex gel phantoms by varying *G*_*diff*_. The other imaging parameters, TR (4000 ms) and TE (75 ms), diffusion gradient direction = R/L, FOV = 36 × 36 mm^2^, acquisition matrix = 32 × 32, slice thickness = 2 mm and number of repetitions (NEX = 4), were kept the same in all sequences.

### 3.3. Data Analysis:

**Figure 2.**
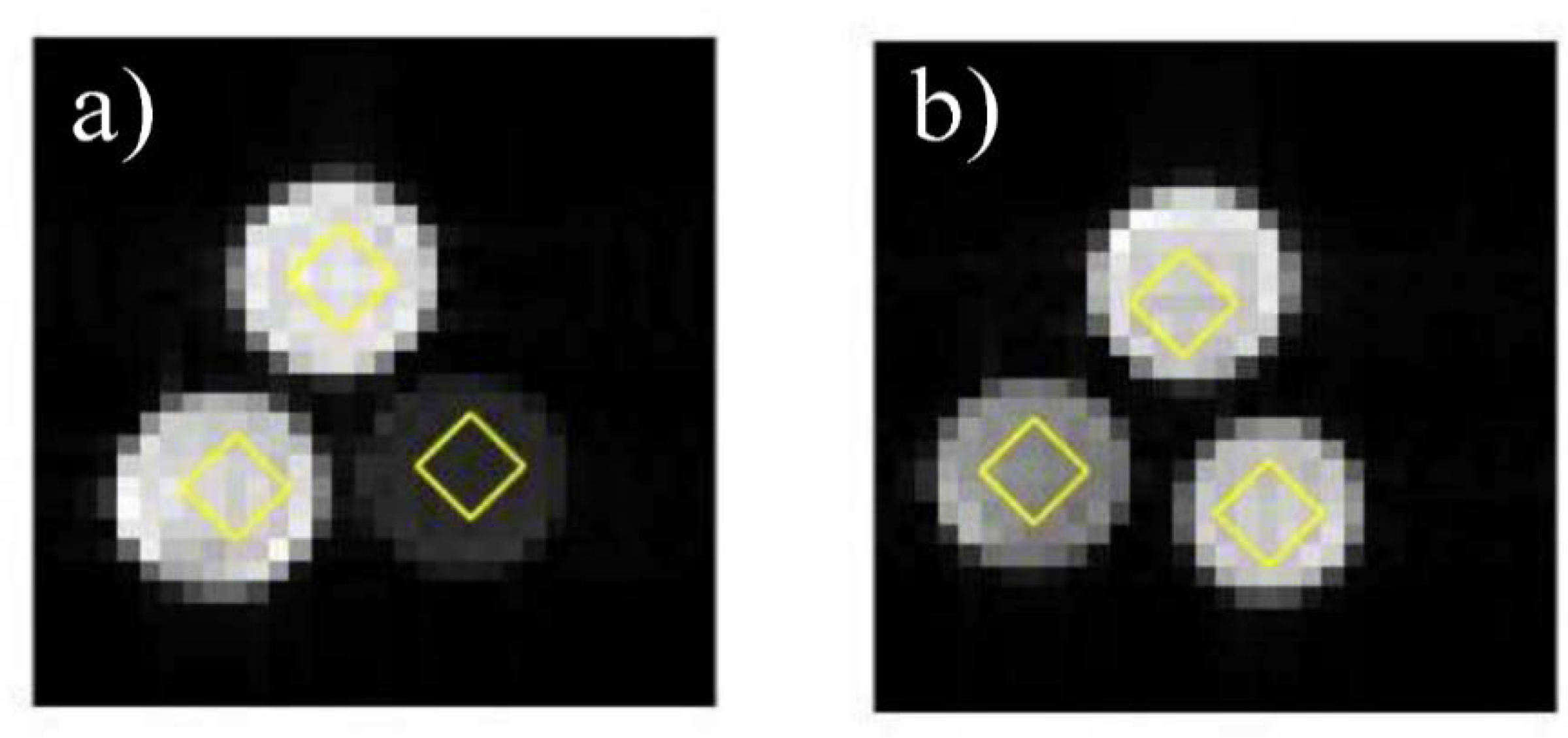
DW images acquired by using the PGSE sequence (Δ = 35 ms and *b* = 0). (**a**) The first Sephadex series: G25–50 (bottom right), G50–50 (bottom left), and G75–50 (top). (**b**) The second Sephadex series: G50–50 (bottom left), G50–80 (bottom right), and G50–150 (top). The rhombus-shaped ROIs indicate the regions used to calculate the mean parameter values.

The DW images acquired by OGSE, PGSE, and PGSTE were first normalized by dividing DW signal, *S*, at each *b*-value by *S*_*0*_. The CTRW model in Equation (7) was fit to the DW images voxel-by-voxel by using an iterative non-linear Levenberg–Marquardt algorithm in MATLAB. To improve the fitting stability and alleviate the degeneracy, *D*_*α,β*_ was first estimated by a mono-exponential model at lower *b*-values, followed by a simultaneous estimation of other parameters with appropriate constraints (0 < *α* ≤ 1 and 0 < *β* ≤ 2) by using all *b*-values [27]. Measurements were made from each quantitative parameter map (*D*_*α,β*_, *α*, and *β*) by computing the mean value over a ~16 mm^2^ region-of-interest (ROI) within each vial of the Sephadex gel. Representative DW images and ROIs are shown in Figure 2.

### 3.4. Monte Carlo Simulations:

Monte Carlo simulations of the time-dependent MR signals were performed with random walkers implemented by using the Monte Carlo Diffusion Simulator of Camino Diffusion MRI Toolkit (UCL) [28,29]. In our simulations, we modeled Sephadex beads with permeable pores as the perpendicular sections of parallelly packed, non-overlapping cylinders with permeable membrane. The permeability was defined as a fixed probability of a random walker stepping through the membrane. Different Sephadex bead sizes and macromolecular exclusion limits were simulated by adjusting the circle radius and permeability as detailed below.

Random walkers were randomly seeded inside and between the hexagonally packed 3D parallel cylinders with permeable membranes; and updated the positions following the rules described in a previous study [28]. Phase change of each random walker was calculated under the custom-specific diffusion gradients. The synthetic DWI signals were then generated by summing the contributions from all random walkers at the echo time. To investigate the effect of varying microstructure scale and membrane permeability on the anomalous diffusion signal behavior and its time dependency, two sets of simulation data were generated to simulate the two Sephadex gel series in the MRI experiments. In the first simulation dataset, substrates with different permeability (*p* = 0.1%, 0.2%, and 0.4%) were chosen with fixed cylinder radius (*r* = 8 μm). In the second simulation dataset, substrates with different cylinder radii (*r* = 6 μm, 7 μm, and 8 μm) were selected with fixed membrane permeability (*p* = 0.2%). Synthetic DWI signals were generated with diffusion gradients that were designed to be perpendicular to the cylinder long axes. For each substrate, three different synthetic OGSE signals were simulated with the oscillating diffusion gradients at three Δ_eff_ values (3.33, 5, and 10 ms). Eleven different synthetic PGSE/PGSTE signals were simulated with Stejskal–Tanner diffusion gradients at eleven Δ_eff_ (25, 30, 35, 40, 45, 50, 60, 70, 80, 90, and 100 ms).

All simulations were performed on an 8-core Intel i7–2600 CPU with 100,000 random walkers and 20,000 time-steps with intrinsic diffusivity of 2 × 10^−3^ mm^2^/s, intracellular volume ratio of 0.5 and 7 *b*-values (0, 200, 500, 1000, 1500, 3000, and 6000 s/mm^2^). The normalized simulated signal intensities over all *b*-values were fit to Equation (7) with the same fitting procedure as for the experimental data.

## 4. Results

**Figure 3.**
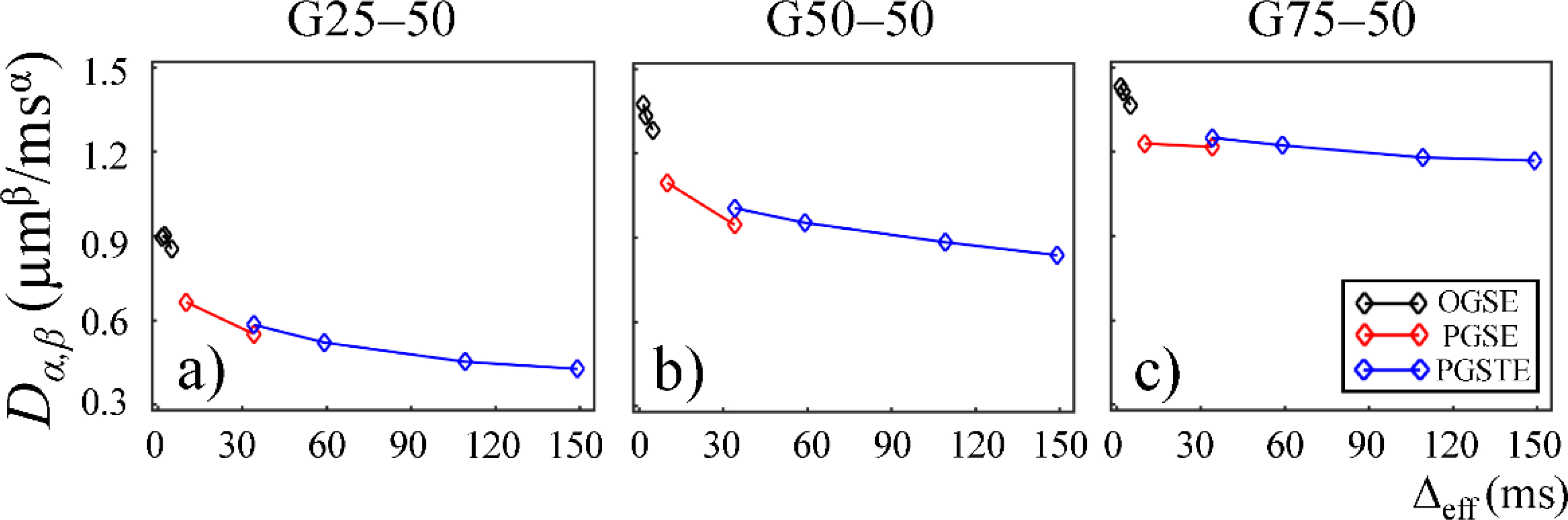
Plots of *D*_*α,β*_ versus effective diffusion time, Δ_eff_, for gels G25–50 (**a**) and G50–50 (**b**), and G75–50 (**c**) with increased macromolecular exclusion limit. The data acquired by using the OGSE, PGSE, and PGSTE pulse sequences are marked in black, red, and blue, respectively.

The CTRW parameter, *D*_*α,β*_, obtained from the first Sephadex gel series was plotted as a function of Δ_eff_ in Figure 3. Two trends were observed. First, a downward trend reaching a plateau was seen for all gels, suggesting increased hinderance at longer diffusion times. Second, the gels with larger pore sizes (G50–50 and G75–50 in Figures 3b,c) exhibited higher *D*_*α,β*_ values at all diffusion times.

**Figure 4.**
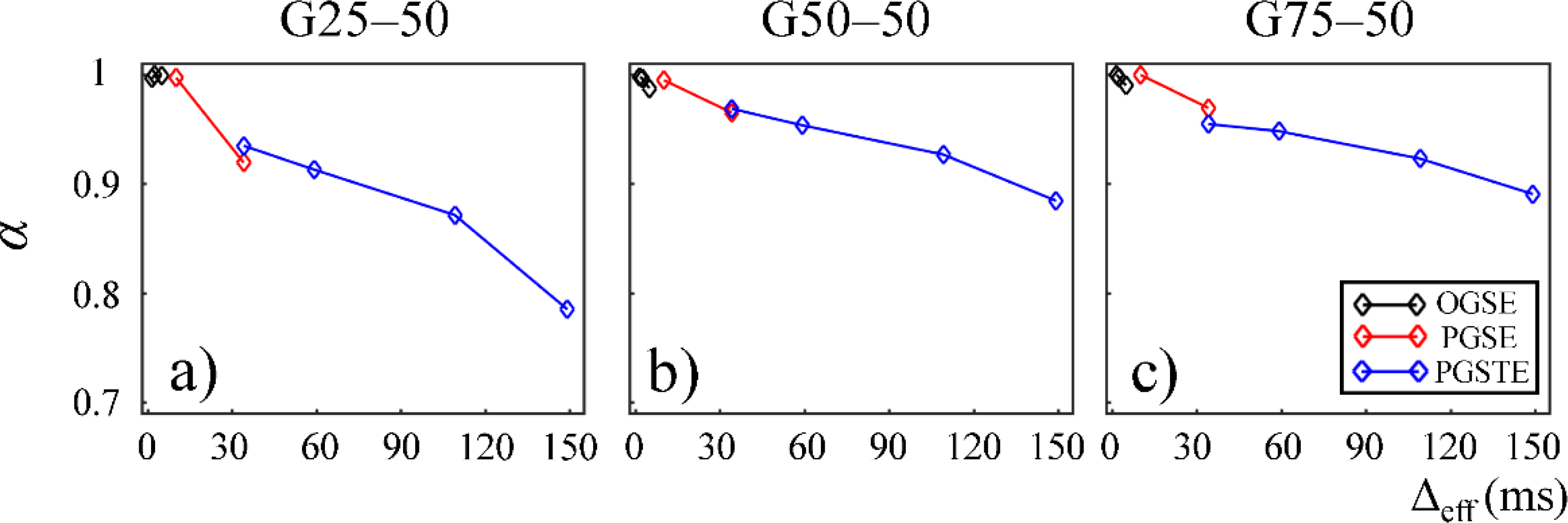
Plots of temporal fractional order (*α*) versus effective diffusion time, Δ_eff_, for gels G25–50 (**a**) and G50–50 (**b**), and G75–50 (**c**). The data acquired by using the OGSE, PGSE, and PGSTE pulse sequences are marked in black, red, and blue, respectively.

**Figure 5.**
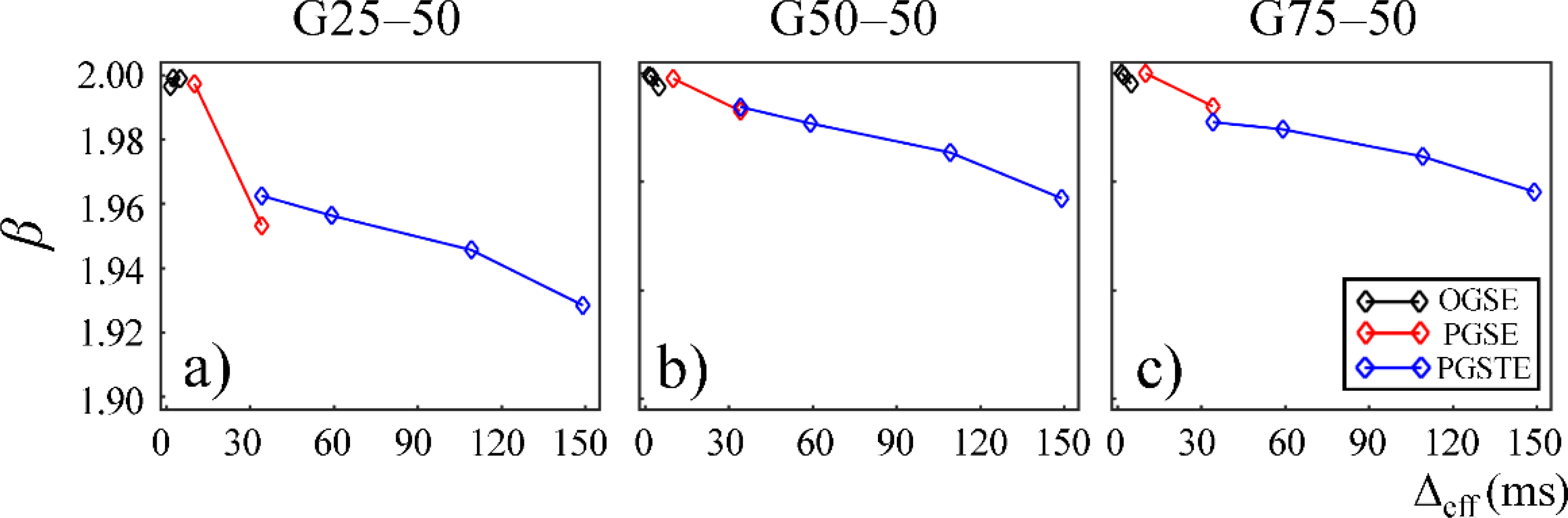
Plots of spatial fractional order (*β*) versus effective diffusion time, Δ_eff_, for gels G25–50 (**a**) and G50–50 (**b**), and G75–50 (**c**). The data acquired by using the OGSE, PGSE, and PGSTE pulse sequences are marked in black, red, and blue, respectively.

In Figures 4 and 5, the fractional order time and space derivatives, *α* and *β*, were plotted against Δ_eff_, respectively. *α* and *β* exhibited a similar trend to each other. For all the gels, as Δ_eff_ decreased to 0, *α* and *β* values approached to 1 and 2, respectively, indicating that the diffusion signal behavior approaches to the Gaussian regime in the limit of short diffusion times. The gels with larger pore sizes exhibited higher *α* and *β* values (G50–50 and G75–50 in Figures 4a,b and 5a,b), suggesting less deviation from Gaussian diffusion dynamics.

**Figure 6.**
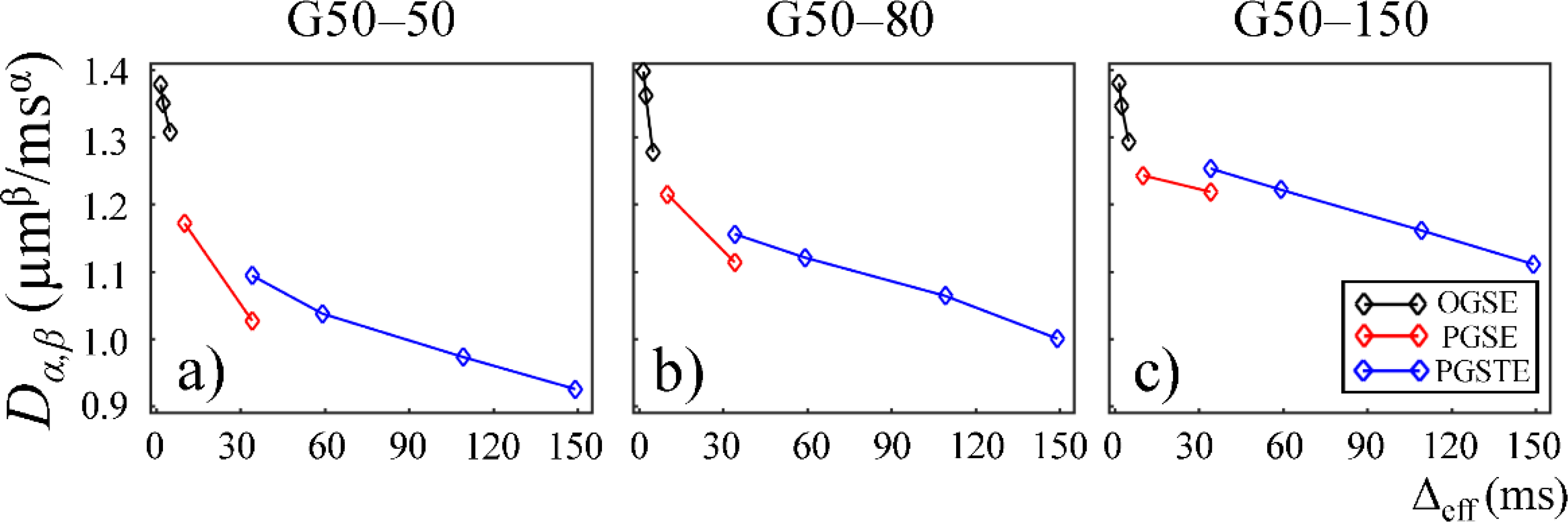
Plot of *D*_*α,β*_ versus effective diffusion time, Δ_eff_, for gels G50–50 (**a**), G50–80 (**b**), and G50–150 (**c**). The data acquired by using the OGSE, PGSE, and PGSTE pulse sequences are marked in black, red, and blue, respectively.

In Figure 6, *D*_*α,β*_ is plotted as a function of Δ_eff_ for the second Sephadex gel series, G50–50, G50–80, and G50–150. Similar to the first Sephadex gel series, *D*_*α,β*_ followed a downward trend reaching a plateau for all the Sephadex gels. G50–50, G50–80, and G50–150 in Figure 6a–c show similar *D*_*α,β*_ values at short diffusion times. However, at longer diffusion times, the gels with larger bead sizes (G50–80 and G50–150 in Figures 6b,c) exhibited higher *D*_*α,β*_ values, similar to what is shown in Figure 3.

**Figure 7.**
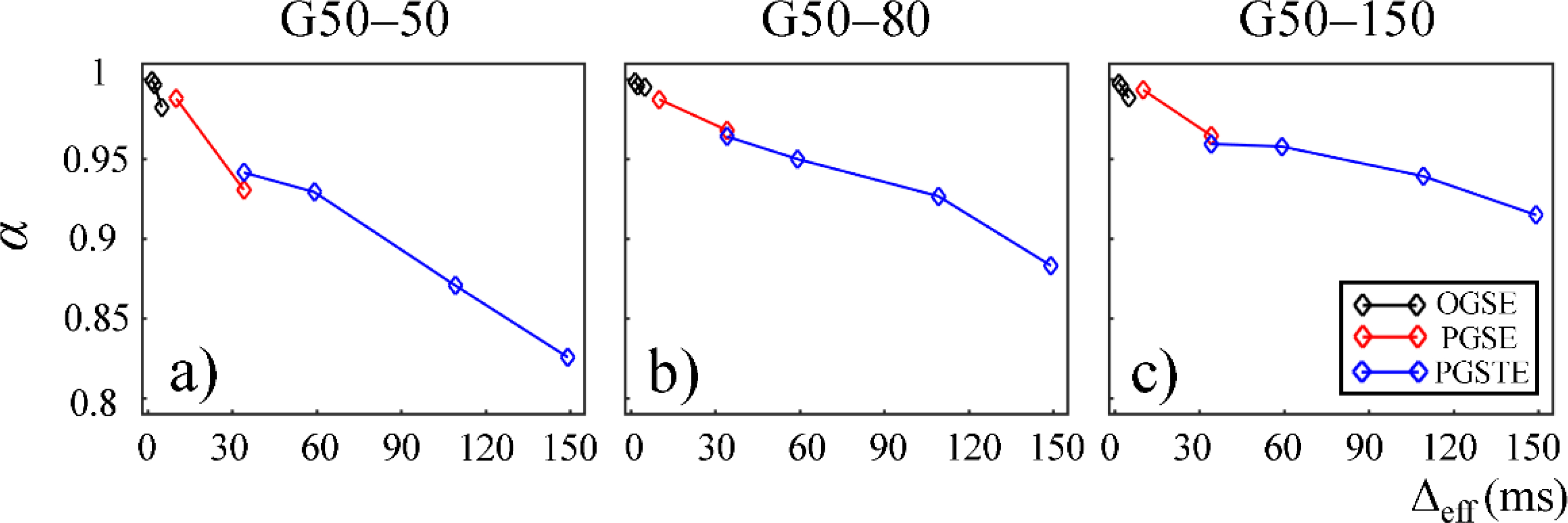
Plot of temporal fractional order (*α*) versus effective diffusion time, Δ_eff_, for gels G50–50 (**a**), G50–80 (**b**), and G50–150 (**c**). The data acquired by using the OGSE, PGSE, and PGSTE pulse sequences are marked in black, red, and blue, respectively.

**Figure 8.**
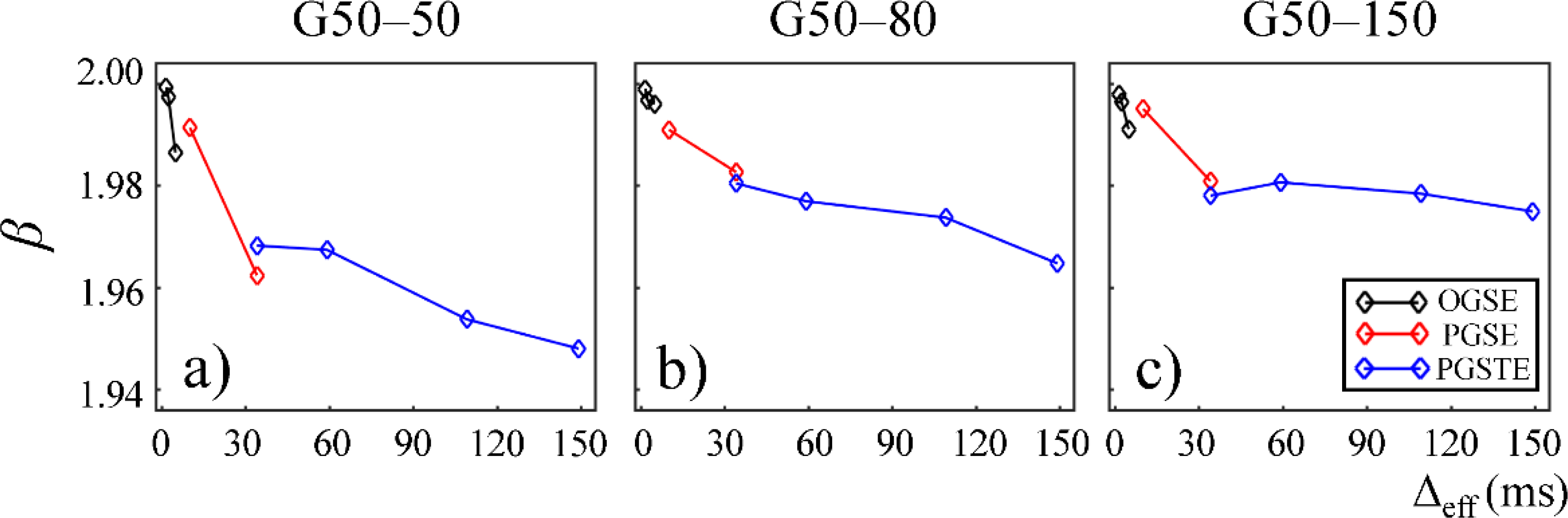
Plot of spatial fractional order (*β*) versus effective diffusion time, Δ_eff_, for gels G50–50 (**a**), G50–80 (**b**), and G50–150 (**c**). The data acquired by using the OGSE, PGSE, and PGSTE pulse sequences are marked in black, red, and blue, respectively.

In Figures 7 and 8, *α* and *β* are plotted against Δ_eff_ for gels in the second Sephadex series (G50–50, G50–80, and G50–150). Similar to the first Sephadex gel series, *α* and *β* showed a decreasing trend against Δ_eff_ in all gels. As the dry bead size increased, higher *α* and *β* values were observed in general. 2*α*/*β* < 1 was observed in all Sephadex gels, indicating the diffusion dynamics fell into sub-diffusion regime.

**Figure 9.**
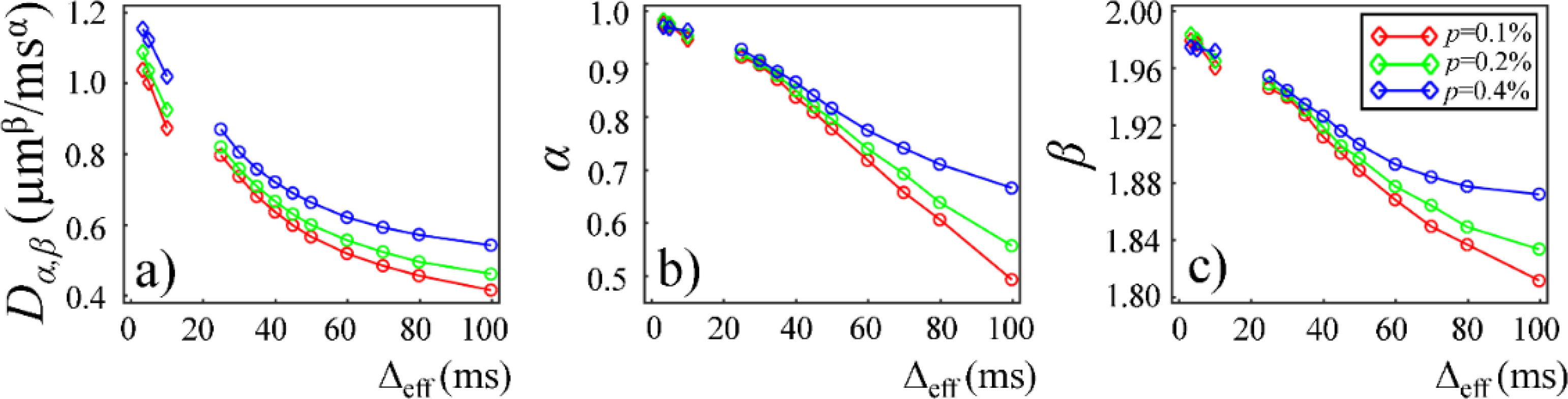
Plots of *D*_*α,β*_ (**a**), *α* (**b**), and *β* (**c**) versus Δ_eff_ obtained from the Monte Carlo simulations with fixed *r* = 8 μm and varying *p* of 0.1% (red), 0.2% (green) and 0.4% (blue). The rhombi and circles represent the simulation results with oscillating diffusion gradient (OGSE) and Stejskal–Tanner diffusion gradient (PGSE/PGSTE), respectively.

**Figure 10.**
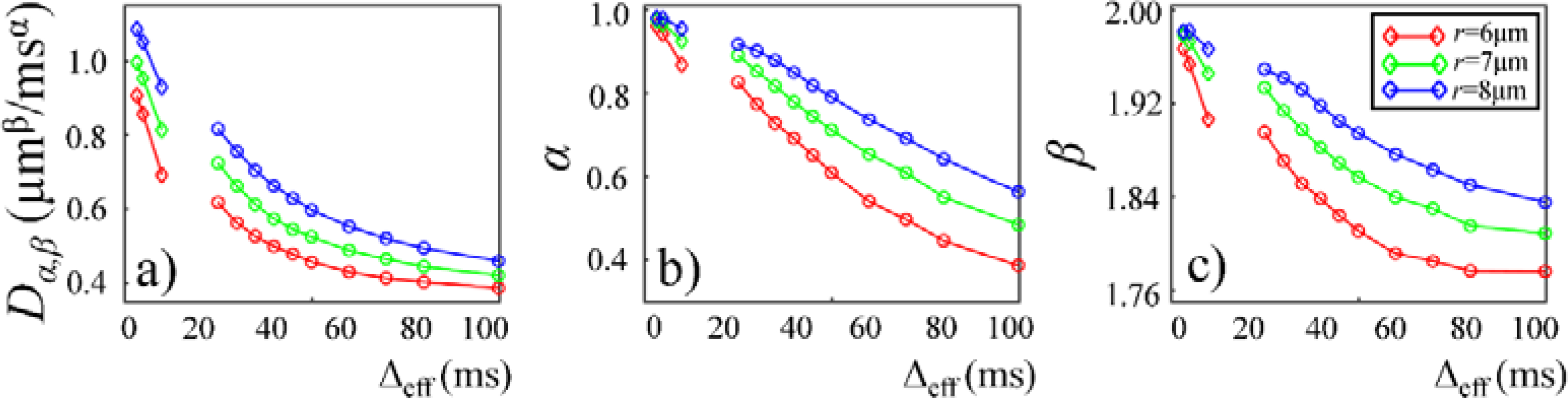
Plots of *D*_*α,β*_ (**a**), *α* (**b**), and *β* (**c**) versus Δ_eff_ from Monte Carlo simulation with fixed *p* = 0.2% and varying *r* of 6 μm (red), 7 μm (green) and 8 μm (blue). The rhombi and circles represent simulation results with oscillating diffusion gradient (OGSE) and Stejskal–Tanner diffusion gradient (PGSE/PGSTE), respectively.

Plots in Figures 9 and 10 show the time-dependent changes observed in the CTRW parameters in the Monte Carlo simulations. In the first simulation dataset (fixed *r* and varying *p* of 0.1%, 0.2%, and 0.4%), the CTRW parameters, *D*_*α,β*_ (Figure 9a), *α* (Figure 9b), and *β* (Figure 9c), yielded higher values in the data with higher permeability. In the second simulation dataset (fixed *p* and varying *r* of 6, 7, and 8 μm), the simulated data with a larger cylinder radius yielded higher *D*_*α,β*_ (Figure 10a), *α* (Figure 10b), and *β* (Figure 10c). The simulation results exhibited a good agreement with experimental results. In both simulations, *D*_*α,β*_, *α*, and *β* exhibited a monotonically decreasing trend.

## 5. Discussion

By employing OGSE, PGSE, and PGSTE sequences to span a broad range of diffusion times, we investigated the diffusion time dependency of the CTRW parameters in Sephadex gel phantoms and correlated our results with Monte Carlo simulations. We observed a monotonic decrease in the CTRW parameters, *D*_*α,β*_, *α*, and *β*, as the diffusion time increased. Our Monte Carlo simulations exhibited a similar trend with the experimental results. To the best of our knowledge, this is the first study which investigates the time dependency behavior of the CTRW model over a wide range of diffusion times using a multi-sequence acquisition scheme.

In the classical mono-exponential model, where water molecules diffuse freely without hinderance and restriction, the MR signal attenuation function is concisely characterized by a commonly used exponential function, exp(−*bD*). In the CTRW model, *α* and *β* quantitatively describe the deviation of diffusion dynamics from the mono-exponential decay [30]. At short diffusion times where MSD is much smaller than the obstacle scales, the water molecules can diffuse freely in all directions, leading to a process that follows Gaussian dynamics. As expected, at this short diffusion time extreme, *α* and *β* values approached to 1 and 2, respectively, while *D*_*α,β*_ converged to the diffusion coefficient of pure water, *D*_*0*_. These outcomes were clearly observed in our experimental data and confirmed in Monte Carlo simulations. In contrast, the long diffusion time provides water molecules a greater opportunity to explore the heterogeneity of the surrounding environment, resulting in reduced *α* and *β*. The increased hinderance and restriction experienced by water molecules at long diffusion times yielded reduced *D*_*α,β*_, *α*, and *β* values, which allows us to infer information on microstructures and micro-environment.

The lower *D*_*α,β*_ values observed in the gels with smaller pore sizes in the first Sephadex gel series (Figure 3a,b) is consistent with the general belief that diffusion coefficient is lower in materials with increased micro-structural barriers [31]. Sephadex gels with larger dry bead sizes exhibited similar *D*_*α,β*_ at low diffusion times, but higher *D*_*α,β*_ at long diffusion times (Figure 6b,c), suggesting that the influence of microstructure scale on diffusion dynamics is more evident at longer diffusion times.

The CTRW model incorporates temporal and spatial diffusion heterogeneities through fractional order time and space parameters, *α* and *β*, respectively. The *α* parameter describes the likelihood of water molecules to be “trapped” or “released” in complex materials, which reflects temporal heterogeneity of diffusion process. The *β* parameter is mathematically equivalent to the heterogeneity parameter in the stretched-exponential model [32], which has been linked with the non-Gaussian distribution of diffusion displacement and shown to be related to intravoxel spatial heterogeneity [18]. In our experimental and simulation results, the lower permeability and smaller microstructural scale of the structural barriers led to a higher likelihood for water molecules to interact with the surrounding structures. This explains the lower *α* and *β* values observed in Sephadex gels with lower macromolecular exclusion limit (Figures 4a and 5a) and smaller bead diameter (Figures 7a and 8a). The experimental observations were further reinforced by the simulation results with lower permeability (Figure 9a) and smaller cylinder radius (Figure 10a).

In this study, some discontinuities were observed in the parameter values when different pulse sequences were employed. For example, *D*_*α,β*_ values obtained from the experiments performed with the PGSTE were higher than those observed by the PGSE at Δ_eff_ = 35 ms (Figures 3 and 6). Also, *α* and *β* values estimated from the data acquired with the PGSE at Δ = 11 ms were higher than those with the OGSE at Δ_eff_ = 5 ms in the first Sephadex series, as shown in Figures 4 and 5. The root cause of the discontinuities is unknown and requires further investigation. Nevertheless, the overall monotonic trend across a broad diffusion time range is consistent in all CTRW parameters. Two Sephadex gel series with varying pore size or bead diameter exhibited the same monotonic trend, which was consistent with the trends revealed by the Monte Carlo simulations.

The accuracy of our simulation depends upon the Monte Carlo Diffusion Simulator of Camino Diffusion MRI Toolkit. The assumption is that this simulator is capable of simulating diverse diffusion processes across a broad range of environments (i.e., substrates), from simple to exceedingly complex. In our simulations, the varying radius and permeability were employed to mimic the varying dry bead diameter and macromolecular exclusion limit of the Sephadex gels, respectively. Although we did not attempt to explicitly evaluate the accuracy and precision of the simulations in this study, a previous study [28] has illustrated the accuracy of a similar simulation approach. Furthermore, the accuracy was likely enhanced by the large number of random walkers (100,000) and time steps (20,000) employed in our study.

Varying diffusion time provides a viable approach to investigating the length scale of tissue microstructures using water diffusion as a probe [10]. This has a direct impact on investigating a range of clinical problems. For example, Lemberskiy et al. [33] utilized time dependent MD and FA for prostate cancer grading while Iima et al. [34] distinguished malignant from benign head and neck tumors by using time dependent apparent diffusion coefficient. Several other studies investigated the time dependency of alternative diffusion models [12,35]. For example, Iima et al. [12] observed significant time dependence of IVIM and DKI parameters in breast cancer and hepatocellular carcinoma xenograft; and Zhou et al. [35] reported time-dependence of the parameters in a fractional order calculus model in the human brain. Although the present study did not focus on a specific clinical problem, the results from the phantom study provided a useful guide to future investigations involving pathological tissue specimens, animal models, and clinical patients.

Our study has several limitations. First, the longest Δ_eff_ in our experiments was limited to 149.17 ms. This was largely due to the inadequate signal-to-noise ratio in the PGSTE acquisition. Additionally, a moderate TE of 75 ms was chosen to match the TE in the OGSE acquisition, thereby mitigating the potential issue with the TE-dependence in diffusion characterization, which can be particularly evident in a multi-compartmental environment [36–38]. If the PGSTE sequence is employed alone without the need to match parameters in other sequences, then studies on diffusion time dependency at long diffusion time regime can be conducted with a shorter TE. Second, our simulations did not cover the low diffusion time regime (e.g., < 3.33 ms for OGSE and < 25 ms for PGSE/PGSTE). This was because the limited total step size of 20,000 could lead to unstable results at shorter diffusion times. Optimized algorithms and more powerful computational platforms may help overcome this limitation. Third, although significant time dependency of CTRW model parameters was observed in this study, the analytical expressions [39] of this time dependency in a multi-compartmental environment requires further investigation. Finally, Sephadex gel phantoms provide a simple diffusion environment with spherical beads and permeable pores. Although they helped provide valuable insights into understanding of the complex diffusion processes, their limitations in adequately mimicking actual biological tissue structures must be recognized.

## 6. Conclusions

We have investigated time dependency of the CTRW model parameters in Sephadex gel phantoms across a broad range of diffusion times by using a set of pulse sequences comprising OGSE, PGSE, and PGSTE. We have experimentally observed monotonic decreases in *D*_*α,β*_, *α*, and *β* as the diffusion time increased. These experimental results were reinforced by the Monte Carlo simulations. The present study provides valuable insights into probing microstructures by characterizing the time dependency of the CTRW model parameters, paving the way for future investigations on biological systems.

## Data Availability

Data available on request.
